# Safety, Efficacy, and Ill Intent: Examining COVID-19 Vaccine Perceptions among the New Undervaccinated Moveable Middle in a U.S. Cohort, October 2022

**DOI:** 10.3390/vaccines11111665

**Published:** 2023-10-31

**Authors:** Rachael Piltch-Loeb, Kate Penrose, Eva Stanton, Angela M. Parcesepe, Yanhan Shen, Sasha A. Fleary, Denis Nash

**Affiliations:** 1 Department of Environmental Occupational and Geospatial Health Sciences, Graduate School of Public Health and Health Policy, City University of New York (CUNY), New York, NY 10027, USA; 2 Institute for Implementation Science in Population Health (ISPH), City University of New York (CUNY), New York, NY 10027, USA; 3 Emergency Preparedness Research Evaluation and Practice Program, Harvard T.H. Chan School of Public Health, Boston, MA 02120, USA; 4 Department of Maternal and Child Health, Gillings School of Global Public Health, University of North Carolina at Chapel Hill, Chapel Hill, NC 27516, USA; 5 Carolina Population Center, University of North Carolina at Chapel Hill, Chapel Hill, NC 27516, USA; 6 Department of Epidemiology and Biostatistics, Graduate School of Public Health and Health Policy, City University of New York (CUNY), New York, NY 10027, USA; 7 Department of Community Health and Social Sciences, Graduate School of Public Health and Health Policy, City University of New York (CUNY), New York, NY 10027, USA

**Keywords:** COVID-19, vaccine hesitancy, undervaccinated, safety, efficacy

## Abstract

Individuals who received their primary vaccine series only (with no subsequent booster) may be a new type of “moveable middle” given their receipt of the original COVID-19 vaccination. One population within the moveable middle for whom tailored interventions may be needed is individuals with common mental disorders (CMD). The purpose of this paper is to understand the vaccine perceptions among this new moveable middle—the undervaccinated—and within the undervaccinated to examine the extent to which COVID-19 vaccine perceptions and motivations differ among those with and without symptoms of CMD. Using data from the CHASING COVID Cohort, we examine the relationship between vaccination status, CMD, and vaccine perceptions in the undervaccinated. Among 510 undervaccinated participants who had completed the primary vaccine series but were not boosted, the most common reasons for undervaccination focused on efficacy (not seeing a need for an additional dose, 42.4%; there not being enough evidence that a booster dose is effective, 26.5%; already having had COVID-19, 19.6%). Other concerns were related to safety (long-term side effects, 21.0%; short-term side effects, 17.6%) and logistics (plan to get a booster but haven’t had time yet, 18.8%). Overall, the greatest vaccine concerns (over 30%) for the undervaccinated focused on efficacy and safety issues. Symptoms of depression or anxiety were associated with lower levels of vaccine efficacy and greater safety concerns in adjusted models. The implications of our study are that campaigns that are hoping to maximize vaccination uptake should consider focusing on and emphasizing messaging on efficacy and safety issues.

## 1. Introduction

COVID-19 remains the fourth-leading cause of death in the United States [1]. The bivalent COVID-19 booster significantly protects against death from COVID-19 [2]. Despite the protective nature of the latest vaccine dose, 92% of the adult U.S. population had received at least one dose of the COVID vaccine as of June 2023, only 79% had completed their primary vaccine series, and only 20.5% had received a bivalent booster dose [3]. Current guidance suggests that additional doses of the COVID-19 vaccine will continue to be recommended for the foreseeable future as vaccine effectiveness wanes over time [4]. Low uptake of the bivalent COVID-19 booster may be associated with a variety of factors, including changing risk-benefit calculations, mixed booster messaging, and the phenomenon of “pandemic fatigue” [5,6].

COVID-19 vaccine misinformation has also continued to permeate the information environment, possibly discouraging boosting [7,8,9]. Prior research has found that both exposure to and belief in COVID-19 vaccine misinformation have been associated with increased COVID-19 vaccine hesitancy [10]. A 2020 randomized controlled trial found that exposure to online COVID-19 vaccine misinformation significantly decreased vaccine intention in both the U.S. and the United Kingdom [11]. Further, in a 2021 study of U.S. adults, misinformation exposure was inversely related to the likelihood of vaccination: as exposure to misinformation increased, the likelihood of being vaccinated decreased [12]. Studies that reviewed literature about the vaccine and booster have found that distrust in COVID-19 information and messengers, including the CDC, government, and healthcare system, increased the likelihood of not getting vaccinated.

The COVID-19 Vaccination Uptake Behavioral Science Task Force for the U.S. Centers for Medicare and Medicaid Services developed a framework to assess vaccine hesitancy in 2021 and identified three categories of vaccine uptake: (1) vaccine acceptors, (2) vaccine refusers, and (3) the moveable middle [13]. The moveable middle were the population group that had not made a decision yet as to their vaccine status and represented a population to focus on for vaccine promotion. The Task Force’s report highlighted three behavioral interventions that could help boost vaccine uptake targeted at the moveable middle. These interventions were to make the vaccine easy to access, use social influence to boost motivation, and build trust in vaccine safety.

Individuals who received their primary vaccine series only (with no subsequent booster) may be a new type of “moveable middle” given their receipt of the original COVID-19 vaccination. Further investigation into the vaccine perceptions and motivations of these individuals and how they compare to acceptors (current on all doses) can inform behavioral interventions to increase COVID-19 vaccine uptake and boost.

This paper has two primary aims. First, it seeks to understand vaccine perceptions, including belief in specific types of vaccine/booster misinformation, among this new moveable middle: the undervaccinated. Second, within the undervaccinated, this paper examines the extent to which COVID-19 vaccine perceptions and motivations differ among those with and without symptoms of anxiety and depression (hereafter referred to as common mental health disorders, or CMD). Individuals with mental health disorders, including CMD, are at greater risk for COVID-19 infection, severe complications, and mortality; yet COVID-19 vaccine uptake remains lower among people with anxiety and depression compared to the general population [14,15,16,17,18].

We explore this secondary aim as part of a larger research effort by the authors to advance our understanding of how to most effectively and appropriately support COVID-19 vaccination and boosting among individuals with common mental health disorders and to inform the tailoring of vaccination intervention strategies for the undervaccinated population.

## 2. Materials and Methods

### 2.1. Participants

The Communities, households, and SARS-CoV-2 Epidemiology (CHASING) COVID Cohort study is a national prospective cohort study launched on 28 March 2020, during the emergence of the COVID-19 pandemic in the U.S. We used internet-based strategies to recruit a geographically and socio-demographically diverse cohort of participants ≥18 years old and residing in the U.S. or U.S. territories. Follow-up has occurred approximately quarterly, from March 2020 to October 2022. Additional recruitment and follow-up details are presented elsewhere [19].

For this analysis, we included CHASING COVID Cohort participants who completed the October 2022 assessment, which was the first assessment where questions about vaccine concerns were asked. This study was approved by the Institutional Review Boards of the City University of New York (CUNY) (New York, NY, USA) (protocol 2020-0256).

### 2.2. COVID-19 Vaccination Status and Vaccine Perceptions and Motivations

In 11 rounds of follow-up assessments conducted between December 2020 and October 2022, participants reported whether they had recently received any COVID-19 vaccine doses and, if so, the vaccine manufacturer, vaccination dates, and number of doses received. Participants who received one dose of the single-dose Johnson & Johnson vaccine or two doses of a double-dose vaccine were considered to have completed the primary vaccine series. Between October 2021 and October 2022, those who had completed the primary vaccine series were also asked to report receipt of any booster doses. In the October 2022 assessment, participants who had not reported completion of the primary series or who reported completion of the primary vaccine series but no booster doses by October 2022 were considered "undervaccinated." Those who had completed the primary vaccine series but not been boosted were asked to select from a list of reasons for not having received a booster (Appendix A).

In the October 2022 assessment, participants were asked their level of agreement with eleven vaccine claim statements. Statements were developed by examining current vaccine narratives in the lay press and examining nationally representative surveys. Participants could also select “don’t know” for each statement. Responses to each statement were scored from 0 (disagree) to 2 (agree), with “don’t know” scored as the middle (1) response. Three statements were reverse-scored, such that higher scores imply greater negative perceptions about vaccines.

### 2.3. Household Vaccination Status and Trusted Vaccine Information Sources

As of October 2022, participants were asked to share the COVID-19 vaccination status of household members. Participants were also asked to identify various entities, groups, or individuals whom they trusted for reliable information about the COVID-19 vaccine (Appendix A). Trust in U.S. public health officials was defined as endorsing trust in the Centers for Disease Control and Prevention (CDC), State Health Department, or Local/County/City Health Department. Trust in medical professionals was defined as endorsing trust in one’s personal physician or other healthcare provider/worker.

### 2.4. Symptoms of Anxiety or Depression

Assessed in October 2022, participants with a score of 10 or higher on the seven-item Generalized Anxiety Disorder (GAD-7) Scale were categorized as having moderate to severe symptoms of anxiety [20], while those with a score of 10 or higher on the eight-item Patient Health Questionnaire (PHQ-8) were categorized as having moderate to severe symptoms of depression [21]. We assessed COVID-19 vaccine perceptions and motivations among individuals with moderate to severe symptoms of anxiety or depression, given the well-documented comorbidity of anxiety and depression in the literature [22].

### 2.5. Sociodemographic Characteristics

Age, gender, race/ethnicity, education, and household annual income were collected at baseline between March 2020 and July 2020. Geographic region, urban vs. rural zip code designation, employment status, children under 18 in the household, and health insurance status were based on the most up-to-date data prior to starting the analysis (October 2022).

### 2.6. Statistical Analysis

Using data on all cohort participants who completed the October 2022 assessment, we first conducted chi-square tests to compare those who were undervaccinated to those who were fully vaccinated and boosted by the participants’ characteristics. We used bar charts to illustrate reasons endorsed by more than 10% of participants for not getting boosted, as well as the proportion of undervaccinated participants who agreed with each vaccine perception statement. We then conducted an exploratory factor analysis (EFA) using the iterated principal axis factoring method with three factors and varimax rotation to assess the underlying structure of the 11 items related to vaccine perceptions. Three factors were specified based on a priori interest in looking at safety, efficacy, and ill intent vaccine constructs. Alpha reliabilities were calculated for each of the three resulting factors, as were mean and median scores for each factor. Participants with an average response score above one (above “Don’t know” and leaning toward endorsing the vaccine concern) were considered to have a “high” score for that particular construct. For each construct, we estimated the proportion of high vaccine concern scores for undervaccinated participants by sociodemographic, household vaccination, trusted vaccine information sources, and symptoms of anxiety or depression. To assess the extent to which characteristics of undervaccinated participants were associated with higher vaccine concerns, crude and adjusted high vaccine concern risk ratios were estimated for each subscale using robust Poisson models. We used robust Poisson models to avoid problems with convergence faced when using log-binomial models. All adjusted models included household vaccination status, trusted information sources, and symptoms of depression or anxiety and were adjusted for age, gender, and race/ethnicity, as well as any additional sociodemographic variables with significant associations in crude models. For all analyses, results were considered statistically significant for alpha levels less than 0.05. Statistical analyses were conducted using SAS 9.4 (Cary, NC, USA).

## 3. Results

Among the 6814 CHASING COVID cohort participants, 4730 (69.4%) completed the October 2022 assessment, including participants from all 50 U.S. states and Puerto Rico. Of these, 3717 (78.6%) were vaccinated and boosted at the time of the assessment, while 572 (12.1%) were undervaccinated and 441 (9.3%) were unvaccinated. Among undervaccinated participants (n = 572), 510 (89.1%) had completed the primary vaccine series but not received any booster doses.

### 3.1. Participant Characteristics Associated with Being Undervaccinated

Compared to boosted participants, larger proportions of younger participants, men, Black/African American and Hispanic participants, participants living in the South, and those living in rural areas were undervaccinated. In addition, larger proportions of individuals without health insurance, participants with less advanced education, those with a household income of less than $50,000, and participants with children in the household were undervaccinated. Living in a household where no other eligible people were known to be vaccinated was significantly associated with lower rates of vaccination and boosting, as was not trusting U.S. public health officials or healthcare providers for reliable information regarding the COVID-19 vaccine. A larger proportion of undervaccinated (vs. boosted) participants reported moderate to severe symptoms of anxiety or depression (Table 1).

### 3.2. COVID-19 Booster Motivations

**Figure 1:**
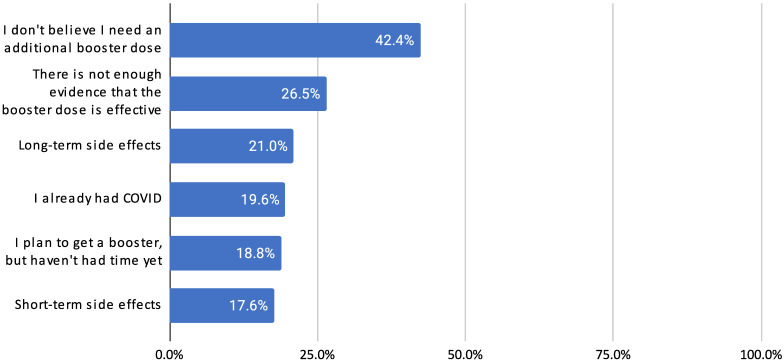
Reasons for not getting boosted reported by more than 10% of participants, among those who had completed the primary COVID-19 vaccine series (N = 510).

**Figure A1:**
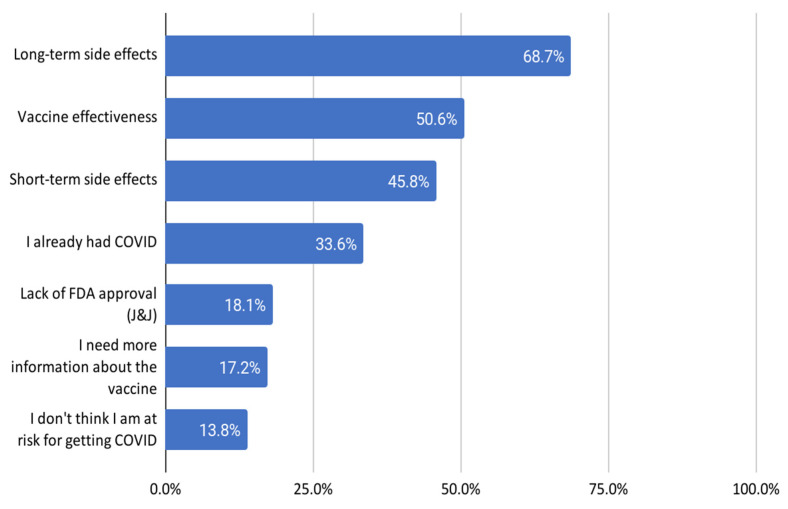
The most common reasons for not getting vaccinated were reported by more than 10% of participants, among CHASING COVID cohort participants who were unvaccinated as of October 2022 (N = 441).

Among 510 undervaccinated participants who had completed the primary vaccine series but not boosted, the most common reasons focused on efficacy (not seeing a need for an additional dose, 42.4%; there not being enough evidence that a booster dose is effective, 26.5%; already having had COVID-19, 19.6%) and somewhat on safety concerns (long-term side effects, 21.0%; short-term side effects, 17.6%) and logistics (plan to get a booster but haven’t had time yet, 18.8%) (Figure 1). Appendix A Figure A1 shows top reasons for all respondents. 

### 3.3. COVID-19 Vaccine Concerns Subscales

**Figure 2:**
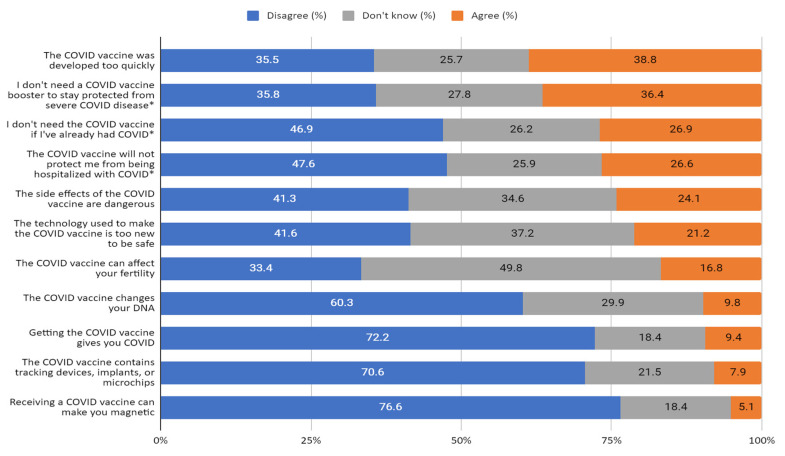
Undervaccinated participant responses to vaccine concern scale items (N = 572). * Reverse scored and reworded to match.

**Figure A2:**
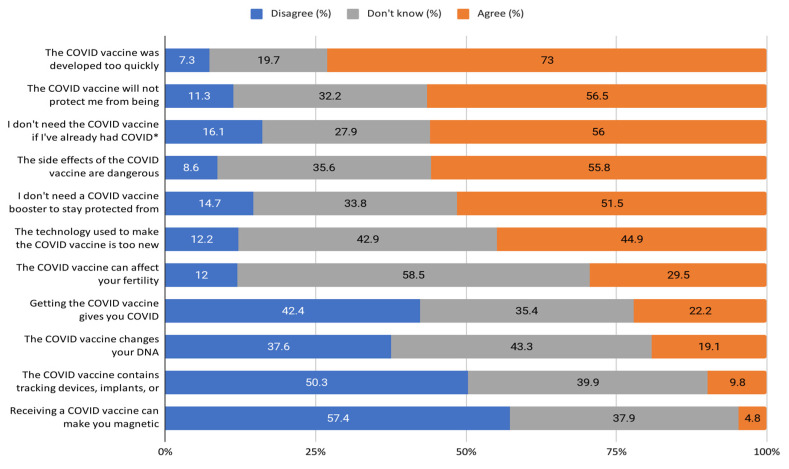
Unvaccinated participant responses to vaccine concern scale items (N = 441).

The vaccine statement with the highest amount of agreement among undervaccinated participants was that the COVID-19 vaccine was developed too quickly (38.8%). More than a third (36.4%) of undervaccinated participants also agreed that a booster wasn’t needed to stay protected from severe COVID, while about a quarter agreed that the COVID vaccine would not protect against hospitalization with COVID (26.6%) or that a vaccine wasn’t needed if one had already had COVID (26.9%). More than one in five undervaccinated participants also agreed that the side effects of the COVID vaccine are dangerous (24.1%) and that the technology used to make the COVID vaccine is too new to be safe (21.2%). On the other hand, less than 10% of undervaccinated participants agreed that the COVID vaccine contains tracking devices or can make a person magnetic (Figure 2). Appendix A Figure A2 shows responses to vaccine concern subscales for unvaccinated participants. 

Vaccine perceptions and their factor loadings for the entire sample (unvaccinated, undervaccinated, and boosted) are found in Table 2. Each item loaded fairly strongly (at least 0.50) on its designated factor, and most had low associations with the other factors. The one exception was the item “The COVID vaccine changes your DNA”, which loaded almost as strongly on the vaccine efficacy concerns construct as on the vaccine ill intent concerns construct (Table 2). As shown in Table 3, mean and median scores for each factor were highest for unvaccinated participants and lowest for boosted participants, with each subscale having high internal consistency (Cronbach’s α for the effiacy concern, safety concern, and ill intent concern subscales were 0.80, 0.85, and 0.83, respectively). Among participants who were undervaccinated, the median safety concern score was highest (1.00 [IQR, 0.25–1.25]), followed by the median efficacy score (0.67 [IQR, 0.33–1.33]). The median concern about ill intent score was the lowest of the three factors measured (0.25 [IQR, 0–0.75]). Table 4, Table 5 and Table 6 are repeated in the Appendix for unvaccinated participants as Table A1, Table A2 and Table A3.

#### 3.3.1. Efficacy Concerns

As shown in Table 4, approximately 34% of undervaccinated participants had vaccine efficacy concern scores greater than 1. Among undervaccinated participants, women or participants who identified as non-binary or whose gender was unknown (ARR: 0.76 [95% CI: 0.62–0.94]), those who trusted public health officials for vaccine information (ARR: 0.41 [95% CI: 0.32–0.51]), and those with moderate to severe symptoms of anxiety or depression (ARR: 0.73 [95% CI: 0.57–0.94]) had a lower risk of having vaccine efficacy concerns. On the other hand, those in a household where no other eligible people were vaccinated or the vaccine status of other household members was unknown (vs. those living in a household where all or some eligible people were vaccinated) were more likely to have vaccine efficacy concerns (ARR: 1.54 [95% CI: 1.19–1.99]).

#### 3.3.2. Safety Concerns

Thirty-two percent of undervaccinated participants had high vaccine safety concern scores (Table 5). Among undervaccinated participants, adjusted risk for vaccine safety concern was lower for participants 18–29 (vs. 50 or older) (ARR: 0.65 [95% CI: 044–0.94]), as well as for those who relied on public health officials for vaccine information (ARR: 0.51 [95% CI: 0.40–0.64]), and those with symptoms of anxiety or depression (ARR: 0.73 [95% CI: 0.55–0.97]). High vaccine safety concern was more common among undervaccinated participants out of the workforce (vs. employed) (ARR: 1.36 [95% CI: 1.03–1.80]).

#### 3.3.3. Concerns about Ill Intent

Approximately 7% of undervaccinated participants had high concerns about vaccine ill intent (Table 6). Among undervaccinated participants, women or participants who identified as non-binary or whose gender was unknown had a lower adjusted risk for vaccine ill intent concern (ARR: 0.52 [95% CI: 0.28–0.94]), as did participants living in the South (vs. the Northeast/Puerto Rico) (ARR: 0.36 [95% CI: 0.18–0.73]) and those with health insurance (ARR: 0.40 [95% CI: 0.21–0.77]). Undervaccinated Black/African American participants (ARR: 2.43 [95% CI: 1.11–5.33]) and participants living in a household where no other eligible people were vaccinated or the vaccine status of other household members was unknown (vs. those living in a household where all or some eligible people were vaccinated) were more likely to have vaccine ill intent concerns (ARR: 2.27 [95% CI: 1.20–4.28]).

## 4. Discussion

In this study, we described undervaccinated individuals in the CHASING COVID Cohort to distinguish their SARS-CoV-2 vaccine concerns. We chose to focus on this group because they may be more likely than those who are unvaccinated to continue with COVID-19 vaccine uptake in the future—they are the new “moveable middle.” Overall, the greatest vaccine concerns for the undervaccinated focused on efficacy and safety issues. When those who completed the primary series but did not receive a booster were asked to identify reasons for not having received a booster, the top three reasons were related to efficacy and included not believing they needed another vaccine dose, perceived efficacy of the booster, and having recently had COVID-19. All of these reasons reflect the perception that the most recent booster does not improve protection against severe COVID-19 beyond the initial vaccine series. Of lower concern were issues related to vaccine safety. These findings suggest that there needs to be a continued effort to focus future vaccine campaigns on why a booster dose supports one’s health, how it works to bolster immunity or protection against severe COVID-19, and whether it is needed regardless of recent infection.

When examining the three constructs of interest: efficacy, safety, and ill intent, we found that over thirty percent of undervaccinated respondents had high efficacy and safety concerns. This was in contrast to only seven percent having concerns related to ill vaccine intentions. Specific pieces of vaccine misinformation that have largely been debunked by trusted health officials (such as the vaccine giving you COVID-19 or the vaccine making you magnetic) seem to be a much lower concern for this moveable middle.

Messengers appeared to have an effect on vaccine uptake, both within and outside of one’s household. Consistent with other studies, individuals may have been influenced by their household in their vaccine perceptions because we found that those who lived in households with no one else who had been vaccinated were more likely to have concerns about vaccine efficacy and ill intent. Our hypothesis is that these familial messengers were having an effect on vaccine decisions. On the other hand, those with trust in messaging from public health officials were less likely to have efficacy or safety concerns. This is consistent with a study by Bennett and colleagues related to boosters that found that trust in the CDC, government, and healthcare system was associated with booster uptake [23]. Building trust in public health and health information with the population remains challenging given the politicized nature of COVID-19 policy, especially in the U.S. However, over two-thirds of undervaccinated participants reported trusting public health officials for COVID-19 vaccine information, a higher portion than those who reported trusting their medical provider for COVID-19 vaccine information. Additional vaccine doses will likely be delivered by routine medical providers, meaning physicians, pharmacists, and nurses will be the primary vaccine risk and benefit communicators. However, it is critical to consider that public health officials may still be effective spokespeople and that household members will remain important influences. There may be opportunities to engage household members collectively to increase vaccine uptake.

One particular interest for our analysis was the relationship between anxiety or depressive symptoms and vaccine perceptions. COVID-19 and its sequelae have had a negative impact on mental health for many US residents. Symptoms of anxiety and depression have been found to relate to vaccine hesitancy, but little has been conducted to explore the extent to which symptoms of anxiety or depression are associated with specific vaccine perceptions. Though symptoms of depression or anxiety were associated with lower vaccine efficacy and safety concerns in adjusted models, they were not associated with the ill intent construct.

Our study is limited in the nature of the associations we are exploring. While most studies look at how vaccine perceptions predict vaccine uptake, we examined the beliefs of individuals who have already made their vaccine choice. This helps inform what constructs may be particularly important to continue shifting COVID-19 vaccine perceptions, but it does not explain whether or not changing the perception will change the behavior. Some individuals who were previously vaccinated may have been compelled to do so for work or social purposes when vaccine mandates are in place. We cannot currently distinguish individuals for whom this was the case. This study is also limited to self-reported vaccination status. Respondents who have chosen to stay engaged in the CHASING COVID Cohort study over time may not be reflective of the general undervaccinated population in the US. Therefore, we focus on comparing those who are undervaccinated with those who are fully vaccinated or unvaccinated. 

Vaccine campaigns that are hoping to maximize efficiency should consider focusing messaging on efficacy and safety issues. Current national efforts appear to focus on COVID-19 health risks to the population, especially older adults, as a talking point to advocate for additional doses of the vaccine [24]. Further promotional materials are focused on addressing general health misinformation. Our results suggest that supplemental information that addresses both efficacy and safety helps people with a variety of circumstances better understand the risks and benefits of continued vaccination. We did not find a difference in the informational needs of older adults compared to the general population. While vaccine misinformation may be of general concern to the public health community, it appears a substantial proportion of individuals have low levels of vaccine misinformation endorsement but remain undervaccinated. This group may benefit from messages that focus on their concerns related to efficacy and safety.

## Figures and Tables

**Table 1:** Participant Characteristics by COVID-19 Vaccination Status as of October 2022.

	Vaccination Status			
	Un- Vaccinated	Under- Vaccinated	Vaccinated and Boosted	Chi-Square *p*-Value for Under- Vaccinated vs. Boosted
	N (col %)	N (col %)	N (col %)	
Total	441	572	3717	
Age (years)				
18–29	107 (24.3%)	160 (28.0%)	763 (20.5%)	<0.0001
30–39	166 (37.6%)	195 (34.1%)	1015 (27.3%)	
40–49	76 (17.2%)	95 (16.6%)	688 (18.5%)	
50+	92 (20.9%)	122 (21.3%)	1251 (33.7%)	
Gender				
Woman	134 (30.4%)	245 (42.8%)	1727 (46.5%)	0.008
Man	304 (68.9%)	319 (55.8%)	1871 (50.3%)	
Non-binary/Transgender/Other	3 (0.7%)	8 (1.4%)	119 (3.2%)	
Race/ethnicity				
Black NH	82 (18.6%)	119 (20.8%)	298 (8.0%)	<0.0001
Hispanic	97 (22.0%)	138 (24.1%)	540 (14.5%)	
White NH	223 (50.6%)	275 (48.1%)	2460 (66.2%)	
Other NH	39 (8.8%)	40 (7.0%)	419 (11.3%)	
Highest level of education				
High school or less	139 (31.5%)	127 (22.2%)	273 (7.3%)	<0.0001
Some college	186 (42.2%)	229 (40.0%)	814 (21.9%)	
College or graduate degree	116 (26.3%)	216 (37.8%)	2630 (70.8%)	
Household income				
<$50,000	271 (61.5%)	311 (54.4%)	1203 (32.4%)	<0.0001
$50,000-$99,999	126 (28.6%)	172 (30.1%)	1203 (32.4%)	
$100,000+	35 (7.9%)	74 (12.9%)	1198 (32.2%)	
Unknown	9 (2.0%)	15 (2.6%)	113 (3.0%)	
Geographic region				
Midwest	81 (18.4%)	95 (16.6%)	667 (17.9%)	<0.0001
Northeast/Puerto Rico	71 (16.1%)	114 (19.9%)	1211 (32.6%)	
South	199 (45.1%)	243 (42.5%)	907 (24.4%)	
West	90 (20.4%)	120 (21.0%)	932 (25.1%)	
Zip code designation				
Rural	202 (45.8%)	202 (35.3%)	1008 (27.1%)	<0.0001
Urban	239 (54.2%)	370 (64.7%)	2709 (72.9%)	
Employment status				
Employed	279 (63.3%)	401 (70.1%)	2614 (70.3%)	0.0001
Out of work	38 (8.6%)	59 (10.3%)	221 (6.0%)	
Homemaker/student/ retired	124 (28.1%)	112 (19.6%)	882 (23.7%)	
Children under the age 18 of in households				
No	157 (35.6%)	269 (47.0%)	2313 (62.2%)	<0.0001
Yes	284 (64.4%)	303 (53.0%)	1404 (37.8%)	
Health insurance				
No/don’t know	91 (20.6%)	110 (19.2%)	255 (6.9%)	<0.0001
Yes	350 (79.4%)	462 (80.8%)	3462 (93.1%)	
Household vaccination status (not including self)				
No eligible people vaccinated/not sure	242 (54.9%)	75 (13.1%)	76 (2.0%)	<0.0001
All or some of the eligible people were vaccinated	129 (29.3%)	397 (69.4%)	2829 (76.1%)	
N/A (live alone)	70 (15.9%)	100 (17.5%)	812 (21.9%)	
Trust U.S. public health officials for reliable information regarding the COVID vaccine				
No/Unknown	251 (56.9%)	206 (36.0%)	484 (13.0%)	<0.0001
Yes	190 (43.1%)	366 (64.0%)	3233 (87.0%)	
Trust your personal physician or other healthcare provider/worker for reliable information regarding the COVID vaccine				
No/Unknown	270 (61.2%)	289 (50.5%)	1085 (29.2%)	<0.0001
Yes	171 (38.8%)	283 (48.5%)	2632 (70.8%)	
Moderate to severe symptoms of anxiety or depression				
No	318 (72.1%)	387 (67.7%)	2921 (78.6%)	<0.0001
Yes	123 (27.9%)	185 (32.3%)	796 (21.4%)	

**Table 2:** Vaccine concern scale items factor loading matrix (EFA) among unvaccinated, undervaccinated, and boosted participants (N = 4730).

Items	Safety Concerns	Efficacy Concerns	Concerns about Ill Intent
The COVID vaccine can affect your fertility	**0.524**	0.270	0.318
I don’t need the COVID vaccine if I’ve already had COVID *	0.285	**0.706**	0.196
The COVID vaccine was developed too quickly	**0.711**	0.333	0.203
The COVID vaccine will not protect me from being hospitalized with COVID *	0.326	**0.565**	0.183
Getting the COVID vaccine gives you COVID	0.301	0.225	**0.572**
The side effects of the COVID vaccine are dangerous	**0.603**	0.363	0.309
The COVID vaccine changes your DNA	0.429	0.269	**0.526**
I don’t need a COVID vaccine booster to stay protected from severe COVID disease *	0.266	**0.759**	0.189
The technology used to make the COVID vaccine is too new to be safe	**0.662**	0.336	0.332
The COVID vaccine contains tracking devices, implants, or microchips	0.219	0.162	**0.789**
Receiving the COVID vaccine can make you magnetic	0.176	0.142	**0.751**

* Questions were reworded and reverse-scored for analysis. Note: Factor loadings representative of a particular factor appear in **boldface**.

**Table 3:** Alpha reliabilities and mean (SD) and median (IQR) COVID-19 vaccine concern subscale scores by participant vaccination status.

	Unvaccinated (N = 441)		Undervaccinated (N = 572)		Vaccinated and Boosted (N = 3717)		Cronbach’s *α*
	Mean (SD)	Median (IQR)	Mean (SD)	Median (IQR)	Mean (SD)	Median (IQR)	
Efficacy concerns	1.41 (0.60)	1.67 (1.00, 2.00)	0.87 (0.65)	0.67 (0.33, 1.33)	0.21 (0.42)	0 (0, 0.33)	0.80
Safety concerns	1.4 (0.48)	1.50 (1.00, 1.75)	0.87 (0.59)	1.00 (0.25, 1.25)	0.30 (0.45)	0 (0, 0.50)	0.85
Concerns about ill intent	0.67 (0.52)	0.75 (0.25, 1.00)	0.38 (0.48)	0.25 (0, 0.75)	0.11 (0.31)	0 (0, 0)	0.83

**Table 4:** High COVID vaccine efficacy concern scores as of October 2022 in the CHASING COVID Cohort for undervaccinated individuals.

	Total N (col%)	N with Efficacy Concerns Score > 1 (col %)	Crude Risk Ratio for Efficacy Concerns Score > 1	Adjusted Risk Ratio for Efficacy Concerns Score > 1 *
Total	572	197		
Age (years)				
18–29	160 (28%)	51 (25.9%)	0.69 (0.52–0.93)	0.86 (0.64–1.15)
30–39	195 (34.1%)	62 (31.5%)	0.69 (0.52–0.92)	0.86 (0.66–1.12)
40–49	95 (16.6%)	28 (14.2%)	0.64 (0.45–0.93)	0.79 (0.57–1.09)
50+	122 (21.3%)	56 (28.4%)	REF	REF
Gender				
Man	245 (42.8%)	103 (52.3%)	REF	REF
Woman or Non-binary/Transgender/Other	327 (57.2%)	94 (47.7%)	0.68 (0.55–0.86)	0.76 (0.62–0.94)
Race/ethnicity				
Black NH	119 (20.8%)	34 (17.3%)	0.73 (0.53–1.00)	0.80 (0.59–1.07)
Hispanic	138 (24.1%)	40 (20.3%)	0.74 (0.55–1.00)	0.88 (0.65–1.19)
White NH	275 (48.1%)	108 (54.8%)	REF	REF
Other NH	40 (7%)	15 (7.6%)	0.95 (0.62–1.46)	0.91 (0.62–1.34)
Highest level of education				
High school or less	127 (22.2%)	44 (22.3%)	0.97 (0.72–1.31)	-
Some college	229 (40%)	76 (38.6%)	0.93 (0.72–1.20)	-
College or graduate degree	216 (37.8%)	77 (39.1%)	REF	-
Household income				
<$50,000	311 (54.4%)	100 (50.8%)	0.88 (0.63–1.24)	-
$50,000–$99,999	172 (30.1%)	65 (33%)	1.04 (0.73–1.48)	-
$100,000+	74 (12.9%)	27 (13.7%)	REF	-
Unknown	15 (2.6%)	5 (2.5%)	0.91 (0.42–1.99)	-
Geographic region				
Midwest	95 (16.6%)	32 (16.2%)	0.94 (0.64–1.36)	-
Northeast/Puerto Rico	114 (19.9%)	41 (20.8%)	REF	-
South	243 (42.5%)	80 (40.6%)	0.92 (0.68–1.24)	-
West	120 (21%)	44 (22.3%)	1.02 (0.73–1.43)	-
Zip code designation				
Rural	202 (35.3%)	78 (39.6%)	1.20 (0.96–1.51)	-
Urban	370 (64.7%)	119 (60.4%)	REF	-
Employment status				
Employed	401 (70.1%)	133 (67.5%)	REF	-
Out of work	59 (10.3%)	16 (8.1%)	0.82 (0.53–1.27)	-
Homemaker/student/ retired	112 (19.6%)	48 (24.4%)	1.29 (1.00–1.67)	-
Children under the age of 18 in household				
No	269 (47%)	89 (45.2%)	REF	-
Yes	303 (53%)	108 (54.8%)	1.08 (0.86–1.35)	-
Health insurance				
No/don’t know	110 (19.2%)	45 (22.8%)	REF	-
Yes	462 (80.8%)	152 (77.2%)	0.80 (0.62–1.04)	-
Household vaccination status (not including self)				
No eligible people have been vaccinated/not sure	75 (13.1%)	39 (19.8%)	1.72 (1.32–2.24)	1.54 (1.19–1.99)
All or some of the eligible people were vaccinated	397 (69.4%)	120 (60.9%)	REF	REF
N/A (live alone)	100 (17.5%)	38 (19.3%)	1.26 (0.94–1.68)	1.28 (0.97–1.70)
Trust U.S. public health officials for reliable information regarding the COVID vaccine				
No/Unknown	206 (36%)	118 (59.9%)	REF	REF
Yes	366 (64%)	79 (40.1%)	0.38 (0.30–0.47)	0.41 (0.32–0.51)
Trust your personal physician or other healthcare provider/worker for reliable information regarding the COVID vaccine				
No/Unknown	289 (50.5%)	110 (55.8%)	REF	REF
Yes	283 (49.5%)	87 (44.2%)	0.81 (0.64–1.02)	0.85 (0.69–1.06)
Moderate to severe symptoms of anxiety or depression				
No	387 (67.7%)	147 (74.6%)	REF	REF
Yes	185 (32.3%)	50 (25.4%)	0.71 (0.54–0.93)	0.73 (0.57–0.94)

* Model adjusted for age, gender, race/ethnicity, trust in information sources, anxiety/depression, and other sociodemographic variables significant in crude models.

**Table 5:** High COVID vaccine safety concern scores as of October 2022 in the CHASING COVID Cohort for undervaccinated individuals.

	Total N (col%)	N with Safety Concerns Score > 1 (col%)	Crude Risk Ratio for Safety Concerns Score > 1	Adjusted Risk Ratio for Safety Concerns Score > 1 *
Total	572	182		
Age (years)				
18–29	160 (28%)	38 (20.9%)	0.53 (0.38–0.74)	0.65 (0.44–0.94)
30–39	195 (34.1%)	57 (31.3%)	0.65 (0.48–0.87)	0.79 (0.58–1.09)
40–49	95 (16.6%)	32 (17.6%)	0.75 (0.53–1.05)	0.95 (0.66–1.37)
50+	122 (21.3%)	55 (30.2%)	REF	REF
Gender				
Man	245 (42.8%)	76 (41.8%)	REF	REF
Woman or Non-binary/Transgender/Other	327 (57.2%)	106 (58.2%)	1.04 (0.82–1.33)	1.09 (0.85–1.38)
Race/ethnicity				
Black NH	119 (20.8%)	39 (21.4%)	0.97 (0.71–1.32)	1.12 (0.82–1.52)
Hispanic	138 (24.1%)	37 (20.3%)	0.79 (0.57–1.09)	0.96 (0.69–1.32)
White NH	275 (48.1%)	93 (51.1%)	REF	REF
Other NH	40 (7%)	13 (7.1%)	0.96 (0.60–1.55)	1.00 (0.64–1.56)
Highest level of education				
High school or less	127 (22.2%)	43 (23.6%)	1.04 (0.77–1.42)	-
Some college	229 (40%)	69 (37.9%)	0.93 (0.71–1.22)	-
College or graduate degree	216 (37.8%)	70 (38.5%)	REF	-
Household income				
<$50,000	311 (54.4%)	97 (53.3%)	0.82 (0.59–1.15)	-
$50,000–$99,999	172 (30.1%)	51 (28%)	0.78 (0.54–1.14)	-
$100,000+	74 (12.9%)	28 (15.4%)	REF	-
Unknown	15 (2.6%)	6 (3.3%)	1.06 (0.53–2.10)	-
Geographic region				
Midwest	95 (16.6%)	27 (14.8%)	0.74 (0.50–1.09)	-
Northeast/Puerto Rico	114 (19.9%)	44 (24.2%)	REF	-
South	243 (42.5%)	79 (43.4%)	0.84 (0.63–1.13)	-
West	120 (21%)	32 (17.6%)	0.69 (0.47–1.01)	-
Zip code designation				
Rural	202 (35.3%)	73 (40.1%)	1.23 (0.96–1.56)	-
Urban	370 (64.7%)	109 (59.9%)	REF	-
Employment status				
Employed	401 (70.1%)	115 (63.2%)	REF	REF
Out of work	59 (10.3%)	18 (9.9%)	1.06 (0.70–1.61)	1.17 (0.78–1.76)
Homemaker/student/ retired	112 (19.6%)	49 (26.9%)	1.53 (1.18–1.98)	1.36 (1.03–1.80)
Children under the age of 18 in household				
No	269 (47%)	76 (41.8%)	REF	-
Yes	303 (53%)	106 (58.2%)	1.24 (0.97–1.58)	-
Health insurance				
No/don’t know	110 (19.2%)	34 (18.7%)	REF	-
Yes	462 (80.8%)	148 (81.3%)	1.04 (0.76–1.41)	-
Household vaccination status (not including self)				
No eligible people vaccinated/not sure	75 (13.1%)	30 (16.5%)	1.35 (0.98–1.85)	1.22 (0.87–1.70)
All or some of the eligible people were vaccinated	397 (69.4%)	118 (64.8%)	REF	REF
N/A (live alone)	100 (17.5%)	34 (18.7%)	1.14 (0.84–1.56)	1.09 (0.80–1.48)
Trust U.S. public health officials for reliable information regarding the COVID vaccine				
No/Unknown	206 (36%)	97 (53.3%)	REF	REF
Yes	366 (64%)	85 (46.7%)	0.49 (0.39–0.62)	0.51 (0.40–0.64)
Trust your personal physician or other healthcare provider/worker for reliable information regarding the COVID vaccine				
No/Unknown	289 (50.5%)	97 (53.3%)	REF	REF
Yes	283 (49.5%)	85 (46.7%)	0.89 (0.70–1.14)	0.88 (0.70–1.12)
Moderate to severe symptoms of anxiety or depression				
No	387 (67.7%)	136 (74.7%)	REF	REF
Yes	185 (32.3%)	46 (25.3%)	0.71 (0.53–0.94)	0.73 (0.55–0.97)

* Model adjusted for age, gender, race/ethnicity, trust in information sources, anxiety/depression, and other sociodemographic variables significant in crude models.

**Table 6:** High COVID vaccine ill intent concern scores as of October 2022 in the CHASING COVID Cohort for undervaccinated individuals.

	Total N (col%)	N with Ill Intent Concerns Score > 1 (col%)	Crude Risk Ratio for Ill Intent Concerns Score > 1	Adjusted Risk Ratio for Ill Intent Concerns Score > 1 *
Total	572	40		
Age (years)				
18–29	160 (28%)	17 (42.5%)	2.59 (0.98–6.83)	1.43 (0.48–4.27)
30–39	195 (34.1%)	16 (40%)	2.00 (0.75–5.33)	1.38 (0.50–3.83)
40–49	95 (16.6%)	2 (5%)	0.51 (0.10–2.59)	0.30 (0.07–1.19)
50+	122 (21.3%)	5 (12.5%)	REF	REF
Gender				
Man	245 (42.8%)	25 (62.5%)	REF	REF
Woman or Non-binary/Transgender/Other	327 (57.2%)	15 (37.5%)	0.45 (0.24–0.83)	0.52 (0.28–0.94)
Race/ethnicity				
Black NH	119 (20.8%)	14 (35%)	2.70 (1.29–5.65)	2.43 (1.11–5.33)
Hispanic	138 (24.1%)	9 (22.5%)	1.49 (0.65–3.46)	1.27 (0.54–3.02)
White NH	275 (48.1%)	12 (30%)	REF	REF
Other NH	40 (7%)	5 (12.5%)	2.86 (1.07–7.70)	2.62 (1.01–6.81)
Highest level of education				
High school or less	127 (22.2%)	7 (17.5%)	0.79 (0.33–1.89)	-
Some college	229 (40%)	18 (45%)	1.13 (0.59–2.19)	-
College or graduate degree	216 (37.8%)	15 (37.5%)	REF	-
Household income				
<$50,000	311 (54.4%)	23 (57.5%)	1.09 (0.43–2.78)	-
$50,000–$99,999	172 (30.1%)	11 (27.5%)	0.95 (0.34–2.63)	-
$100,000+	74 (12.9%)	5 (12.5%)	REF	-
Unknown	15 (2.6%)	1 (2.5%)	0.99 (0.12–7.85)	-
Geographic region				
Midwest	95 (16.6%)	6 (15%)	0.51 (0.21–1.29)	0.60 (0.24–1.54)
Northeast/Puerto Rico	114 (19.9%)	14 (35%)	REF	REF
South	243 (42.5%)	13 (32.5%)	0.44 (0.21–0.90)	0.36 (0.18–0.73)
West	120 (21%)	7 (17.5%)	0.48 (0.20–1.13)	0.51 (0.22–1.18)
Zip code designation				
Rural	202 (35.3%)	11 (27.5%)	0.69 (0.35–1.36)	-
Urban	370 (64.7%)	29 (72.5%)	REF	-
Employment status				
Employed	401 (70.1%)	34 (85%)	REF	-
Out of work	59 (10.3%)	3 (7.5%)	0.60 (0.19–1.89)	-
Homemaker/student/ retired	112 (19.6%)	3 (7.5%)	0.32 (0.10–1.01)	-
Children under the age of 18 in household				
No	269 (47%)	16 (40%)	REF	-
Yes	303 (53%)	24 (60%)	1.33 (0.72–2.45)	-
Health insurance				
No/don’t know	110 (19.2%)	18 (45%)	REF	REF
Yes	462 (80.8%)	22 (55%)	0.29 (0.16–0.52)	0.40 (0.21–0.77)
Household vaccination status (not including self)				
No eligible people vaccinated/not sure	75 (13.1%)	10 (25%)	2.04 (1.02–4.04)	2.27 (1.20–4.28)
All or some of the eligible people were vaccinated	397 (69.4%)	26 (65%)	REF	REF
N/A (live alone)	100 (17.5%)	4 (10%)	0.61 (0.22–1.71)	0.74 (0.25–2.20)
Trust U.S. public health officials for reliable information regarding the COVID vaccine				
No/Unknown	206 (36%)	18 (45%)	REF	REF
Yes	366 (64%)	22 (55%)	0.69 (0.38–1.25)	0.87 (0.48–1.59)
Trust personal physician or other healthcare provider/worker for reliable information regarding the COVID vaccine				
No/Unknown	289 (50.5%)	26 (65%)	REF	REF
Yes	283 (49.5%)	14 (35%)	0.55 (0.29–1.03)	0.71 (0.38–1.32)
Moderate to severe symptoms of anxiety or depression				
No	387 (67.7%)	27 (67.5%)	REF	REF
Yes	185 (32.3%)	13 (32.5%)	1.01 (0.53–1.91)	0.83 (0.46–1.52)

* Model adjusted for age, gender, race/ethnicity, trust in information sources, anxiety/depression, and other sociodemographic variables significant in crude models.

## Data Availability

Data are available upon request from the authors.

## Appendix A. Survey Items, Additional Figures and Tables

**Survey items**

1. Do you agree with the following claims about the COVID vaccine?

**Table:**

	Agree	Disagree	Don’t Know
The COVID vaccine can affect your fertility.			
I need the COVID vaccine, even if I’ve already had COVID.			
The COVID vaccine was developed too quickly.			
The COVID vaccine will protect me from being hospitalized with COVID.			
Getting the COVID vaccine gives you COVID.			
The side effects of the COVID vaccine are dangerous.			
The COVID vaccine changes your DNA.			
I need a COVID vaccine booster to stay protected from severe COVID disease.			
The technology used to make the COVID vaccine is too new to be safe.			
The COVID vaccine contains tracking devices, implants, or microchips.			
Receiving a COVID vaccine can make you magnetic.			

2. Which of the following influenced your decision not to get a vaccine? Please select all that apply.
  (a) Short-term side effects
  (b) Long-term side effects
  (c) Vaccine effectiveness
  (d) Whether other people I know also get it
  (e) I think that other people should get it before me
  (f) I need more information about the vaccine
  (g) I already had COVID
  (h) I don’t think I am at risk of getting COVID
  (i) I have a medical condition that prevents me from getting vaccinated
  (j) Issues with accessing a vaccine at a time that works for me
  (k) Issues with accessing a specific vaccine versus the one that is available
  (l) Lack of FDA approval (Johnson & Johnson vaccine)
  (m) Other _____________
  (n) None of the above
3. Since your last survey, which of the following has influenced your decision not to get a booster? Please select all that apply.
  (a) I don’t believe I need an additional booster dose
  (b) There is not enough evidence that the booster dose is effective
  (c) I’m not yet eligible for the booster dose
  (d) I’m not sure if I’m eligible for the booster dose
  (e) Short-term side effects
  (f) Long-term side effects
  (g) Whether other people I know also get it
  (h) I think that other people should get it before me
  (i) I need more information about the booster dose
  (j) I already had COVID
  (k) I recently had COVID
  (l) I don’t think I am at risk of getting COVID
  (m) I have a medical condition that prevents me from getting boosted
  (n) Issues with accessing a booster dose at a time (or venue) that works for me
  (o) Issues with accessing a specific vaccine booster dose versus the one that is available
  (p) Lack of full FDA approval (Johnson & Johnson vaccine)
  (q) I plan to get a booster, but I haven’t had time yet
  (r) I’m worried that there will be fees or other costs if I get the booster
  (s) I’m scared of needles
  (t) Other _____________
  (u) None of the above
4. Who do you trust to give you reliable information regarding the COVID-19 vaccine? Please select all that apply.
  (a) Centers for Disease Control and Prevention (CDC)
  (b) World Health Organization (WHO)
  (c) Surgeon General
  (d) White House
  (e) President
  (f) State Health Department
  (g) Local/County/City Health Department
  (h) Your governor
  (i) Your mayor
  (j) Personal physician
  (k) Other healthcare provider/worker
  (l) Family member
  (m) Close Friend
  (n) Religious leader/clergy
  (o) Food and Drug Administration (FDA)
  (p) Significant other/spouse
  (q) Work colleagues
  (r) News media (e.g., television or print)
  (s) A social media network member’s post (e.g., anyone you are friends with or follow on social media)
  (t) Other: _________

**Table A1:** High COVID vaccine efficacy concern scores as of October 2022 in the CHASING COVID Cohort for unvaccinated individuals.

	Total N (col%)	N with Efficacy Concerns Score > 1 (col%)	Crude Risk Ratio for Efficacy Concerns Score > 1	Adjusted Risk Ratio for Efficacy Concerns Score > 1 *
Total	441	288		
Age (years)				
18–29	107 (24.3%)	64 (22.2%)	0.81 (0.66–0.99)	0.91 (0.75–1.09)
30–39	166 (37.6%)	105 (36.5%)	0.86 (0.72–1.01)	0.92 (0.78–1.08))
40–49	76 (17.2%)	51 (17.7%)	0.91 (0.74–1.11)	1.00 (0.82–1.22)
50+	92 (20.9%)	68 (23.6%)	REF	REF
Gender				
Man	134 (30.4%)	98 (34%)	REF	REF
Woman or Non-binary/Transgender/Other	307 (69.6%)	190 (66%)	0.85 (0.74–0.97)	0.86 (0.76–0.98)
Race/ethnicity				
Black NH	82 (18.6%)	43 (14.9%)	0.77 (0.61–0.96)	0.83 (0.67–1.03)
Hispanic	97 (22%)	67 (23.3%)	1.01 (0.86–1.19)	1.13 (0.97–1.32)
White NH	223 (50.6%)	152 (52.8%)	REF	REF
Other NH	39 (8.8%)	26 (9%)	0.98 (0.77–1.24)	0.96 (0.77–1.19)
Highest level of education				
High school or less	139 (31.5%)	85 (29.5%)	0.85 (0.72–1.02)	-
Some college	186 (42.2%)	120 (41.7%)	0.90 (0.77–1.05)	-
College or graduate degree	116 (26.3%)	83 (28.8%)	REF	-
Household income				
<$50,000	271 (61.5%)	167 (58%)	0.80 (0.65–0.98)	0.91 (0.75–1.09)
$50,000–$99,999	126 (28.6%)	89 (30.9%)	0.92 (0.74–1.13)	0.95 (0.79–1.14)
$100,000+	35 (7.9%)	27 (9.4%)	REF	REF
Unknown	9 (2%)	5 (1.7%)	0.72 (0.39–1.33)	0.92 (0.51–1.68)
Geographic region				
Midwest	81 (18.4%)	50 (17.4%)	1.04 (0.81–1.35)	-
Northeast/ Puerto Rico	71 (16.1%)	42 (14.6%)	REF	-
South	199 (45.1%)	131 (45.5%)	1.11 (0.90–1.38)	-
West	90 (20.4%)	65 (22.6%)	1.22 (0.97–1.54)	-
Zip code designation				
Rural	202 (45.8%)	134 (46.5%)	1.03 (0.90–1.18)	-
Urban	239 (54.2%)	154 (53.5%)	REF	-
Employment status				
Employed	279 (63.3%)	195 (67.7%)	REF	REF
Out of work	38 (8.6%)	17 (5.9%)	0.64 (0.45–0.92)	0.63 (0.45–0.89)
Homemaker/student/ retired	124 (28.1%)	76 (26.4%)	0.88 (0.75–1.03)	0.87 (0.74–1.02)
Children under the age of 18 in households				
No	157 (35.6%)	107 (37.2%)	REF	-
Yes	284 (64.4%)	181 (62.8%)	0.94 (0.81–1.07)	-
Health insurance				
No/don’t know	91 (20.6%)	50 (17.4%)	REF	REF
Yes	350 (79.4%)	238 (82.6%)	1.24 (1.01–1.51)	1.16 (0.95–1.42)
Household vaccination status (not including self)				
No eligible people vaccinated/not sure	242 (54.9%)	172 (59.7%)	1.27 (1.07–1.51)	1.18 (1.00–1.39)
All or some of the eligible people were vaccinated	129 (29.3%)	72 (25%)	REF	REF
N/A (live alone)	70 (15.9%)	44 (15.3%)	1.13 (0.89–1.43)	1.04 (0.84–1.31)
Trust U.S. public health officials for reliable information regarding the COVID vaccine				
No/Unknown	251 (56.9%)	193 (67%)	REF	REF
Yes	190 (43.1%)	95 (33%)	0.65 (0.56–0.76)	0.66 (0.56–0.77)
Trust your personal physician or other healthcare provider/worker for reliable information regarding the COVID vaccine				
No/Unknown	270 (61.2%)	171 (59.4%)	REF	REF
Yes	171 (38.8%)	117 (40.6%)	1.08 (0.94–1.24)	1.13 (0.99–1.29)
Moderate to severe symptoms of anxiety or depression				
No	318 (72.1%)	213 (74%)	REF	REF
Yes	123 (27.9%)	75 (26%)	0.91 (0.77–1.07)	1.01 (0.85–1.19)

* Model adjusted for age, gender, race/ethnicity, trust in information sources, anxiety/depression, and other sociodemographic variables significant in crude models.

**Table A2:** High COVID vaccine safety concern scores as of October 2022 in the CHASING COVID Cohort for unvaccinated individuals.

	Total N (col%)	N with Safety Concerns Score > 1 (col%)	Crude Risk Ratio for Safety Concerns Score > 1	Adjusted Risk Ratio for Safety Concerns Score > 1 *
Total	441	316		
Age (years)				
18–29	107 (24.3%)	69 (21.8%)	0.80 (0.67–0.95)	0.85 (0.72–1.01)
30–39	166 (37.6%)	115 (36.4%)	0.86 (0.75–0.99)	0.89 (0.78–1.03)
40–49	76 (17.2%)	58 (18.4%)	0.95 (0.81–1.11)	0.99 (0.84–1.18)
50+	92 (20.9%)	74 (23.4%)	REF	REF
Gender				
Man	134 (30.4%)	90 (28.5%)	REF	REF
Woman or Non-binary/Transgender/Other	307 (69.6%)	226 (71.5%)	1.10 (0.96–1.26)	1.07 (0.94–1.22)
Race/ethnicity				
Black NH	82 (18.6%)	52 (16.5%)	0.87 (0.73–1.05)	0.93 (0.78–1.11)
Hispanic	97 (22%)	74 (23.4%)	1.05 (0.92–1.20)	1.12 (0.97–1.29)
White NH	223 (50.6%)	162 (51.3%)	REF	REF
Other NH	39 (8.8%)	28 (8.9%)	0.99 (0.80–1.22)	0.96 (0.78–1.18)
Highest level of education				
High school or less	139 (31.5%)	100 (31.6%)	1.01 (0.86–1.17)	-
Some college	186 (42.2%)	133 (42.1%)	1.00 (0.86–1.16)	-
College or graduate degree	116 (26.3%)	83 (26.3%)	REF	-
Household income				
<$50,000	271 (61.5%)	189 (59.8%)	0.84 (0.71–1.00)	0.88 (0.75–1.03)
$50,000–$99,999	126 (28.6%)	93 (29.4%)	0.89 (0.74–1.07)	0.92 (0.78–1.09)
$100,000+	35 (7.9%)	29 (9.2%)	REF	REF
Unknown	9 (2%)	5 (1.6%)	0.67 (0.37–1.23)	0.68 (0.37–1.25)
Geographic region				
Midwest	81 (18.4%)	56 (17.7%)	0.98 (0.80–1.21)	-
Northeast/ Puerto Rico	71 (16.1%)	50 (15.8%)	REF	-
South	199 (45.1%)	145 (45.9%)	1.03 (0.87–1.23)	-
West	90 (20.4%)	65 (20.6%)	1.03 (0.84–1.25)	-
Zip code designation				
Rural	202 (45.8%)	146 (46.2%)	1.02 (0.90–1.14)	-
Urban	239 (54.2%)	170 (53.8%)	REF	-
Employment status				
Employed	279 (63.3%)	200 (63.3%)	REF	-
Out of work	38 (8.6%)	26 (8.2%)	0.95 (0.76–1.20)	-
Homemaker/student/ retired	124 (28.1%)	90 (28.5%)	1.01 (0.89–1.15)	-
Children under the age of 18 in households				
No	157 (35.6%)	114 (36.1%)	REF	-
Yes	284 (64.4%)	202 (63.9%)	0.98 (0.87–1.11)	-
Health insurance				
No/don’t know	91 (20.6%)	51 (16.1%)	REF	REF
Yes	350 (79.4%)	265 (83.9%)	1.35 (1.12–1.64)	1.29 (1.06–1.56)
Household vaccination status (not including self)				
No eligible people vaccinated/not sure	242 (54.9%)	186 (58.9%)	1.18 (1.02–1.36)	1.12 (0.97–1.30)
All or some of the eligible people were vaccinated	129 (29.3%)	84 (26.6%)	REF	REF
N/A (live alone)	70 (15.9%)	46 (14.6%)	1.01 (0.82–1.25)	0.99 (0.80–1.23)
Trust U.S. public health officials for reliable information regarding the COVID vaccine				
No/Unknown	251 (56.9%)	188 (59.5%)	REF	REF
Yes	190 (43.1%)	128 (40.5%)	0.90 (0.80–1.02)	0.90 (0.80–1.03)
Trust your personal physician or other healthcare provider/worker for reliable information regarding the COVID vaccine				
No/Unknown	270 (61.2%)	186 (58.9%)	REF	REF
Yes	171 (38.8%)	130 (41.1%)	1.10 (0.98–1.24)	1.07 (0.95–1.20)
Moderate to severe symptoms of anxiety or depression				
No	318 (72.1%)	226 (71.5%)	REF	REF
Yes	123 (27.9%)	90 (28.5%)	1.03 (0.91–1.17)	1.08 (0.94–1.23)

* Model adjusted for age, gender, race/ethnicity, trust in information sources, anxiety/depression, and other sociodemographic variables significant in crude models.

**Table A3:** High COVID concern about ill intent scores as of October 2022 in the CHASING COVID Cohort for unvaccinated individuals.

	Total N (col%)	N with Ill Intent Concerns Score > 1 (col %)	Crude Risk Ratio for Ill Intent Concerns Score > 1	Adjusted Risk Ratio for Ill Intent Concerns Score > 1 *
Total	441	74		
Age (years)				
18–29	107 (24.3%)	13 (17.6%)	0.62 (0.32–1.20)	0.49 (0.26–0.92)
30–39	166 (37.6%)	29 (39.2%)	0.89 (0.53–1.52)	0.74 (0.43–1.26)
40–49	76 (17.2%)	14 (18.9%)	0.94 (0.50–1.77)	0.87 (0.47–1.61)
50+	92 (20.9%)	18 (24.3%)	REF	REF
Gender				
Cis Male	134 (30.4%)	23 (31.1%)	REF	REF
Cis Female or Non-binary/Transgender/Other	307 (69.6%)	51 (68.9%)	0.97 (0.62–1.52)	0.92 (0.60–1.42)
Race/ethnicity				
Black NH	82 (18.6%)	16 (21.6%)	1.45 (0.84–2.52)	1.58 (0.92–2.71)
Hispanic	97 (22%)	25 (33.8%)	1.92 (1.19–3.08)	2.09 (1.32–3.33)
White NH	223 (50.6%)	30 (40.5%)	REF	REF
Other NH	39 (8.8%)	3 (4.1%)	0.57 (0.18–1.78)	0.48 (0.14–1.58)
Highest level of education				
High school or less	139 (31.5%)	23 (31.1%)	1.07 (0.61–1.88)	-
Some college	186 (42.2%)	33 (44.6%)	1.14 (0.68–1.93)	-
College or graduate degree	116 (26.3%)	18 (24.3%)	REF	-
Household income				
<$50,000	271 (61.5%)	43 (58.1%)	0.93 (0.43–2.02)	-
$50,000–$99,999	126 (28.6%)	24 (32.4%)	1.11 (0.49–2.50)	-
$100,000+	35 (7.9%)	6 (8.1%)	REF	-
Unknown	9 (2%)	1 (1.4%)	0.65 (0.09–4.72)	-
Geographic region				
Midwest	81 (18.4%)	9 (12.2%)	0.49 (0.23–1.05)	-
Northeast/Puerto Rico	71 (16.1%)	16 (21.6%)	REF	-
South	199 (45.1%)	37 (50%)	0.83 (0.49–1.39)	-
West	90 (20.4%)	12 (16.2%)	0.59 (0.30–1.17)	-
Zip code designation				
Rural	202 (45.8%)	32 (43.2%)	0.90 (0.59–1.37)	-
Urban	239 (54.2%)	42 (56.8%)	REF	-
Employment status				
Employed	279 (63.3%)	53 (71.6%)	REF	-
Out of work	38 (8.6%)	6 (8.1%)	0.83 (0.38–1.80)	-
Homemaker/student/ retired	124 (28.1%)	15 (20.3%)	0.64 (0.37–1.08)	-
Children under the age of 18 in households				
No	157 (35.6%)	26 (35.1%)	REF	-
Yes	284 (64.4%)	48 (64.9%)	1.02 (0.66–1.58)	-
Health insurance				
No/don’t know	91 (20.6%)	11 (14.9%)	REF	-
Yes	350 (79.4%)	63 (85.1%)	1.49 (0.82–2.71)	-
Household vaccination status (not including self)				
No eligible people vaccinated/not sure	242 (54.9%)	45 (60.8%)	1.33 (0.81–2.20)	1.40 (0.86–2.27)
All or some of the eligible people vaccinated	129 (29.3%)	18 (24.3%)	REF	REF
N/A (live alone)	70 (15.9%)	11 (14.9%)	1.13 (0.56–2.25)	1.07 (0.56–2.06)
Trust U.S. public health officials for reliable information regarding the COVID vaccine				
No/Unknown	251 (56.9%)	52 (70.3%)	REF	REF
Yes	190 (43.1%)	22 (29.7%)	0.56 (0.35–0.89)	0.62 (0.39–0.97)
Trust your personal physician or other healthcare provider/worker for reliable information regarding the COVID vaccine				
No/Unknown	270 (61.2%)	59 (79.7%)	REF	REF
Yes	171 (38.8%)	15 (20.3%)	0.40 (0.24–0.68)	0.45 (0.25–0.79)
Moderate to severe symptoms of anxiety or depression				
No	318 (72.1%)	49 (66.2%)	REF	REF
Yes	123 (27.9%)	25 (33.8%)	1.32 (0.85–2.04)	1.58 (1.04–2.40)

* Model adjusted for age, gender, race/ethnicity, trust in information sources, anxiety/depression, and other sociodemographic variables significant in crude models.
